# Influence of Variety and Storage Time of Fresh Garlic on the Physicochemical and Antioxidant Properties of Black Garlic

**DOI:** 10.3390/foods8080314

**Published:** 2019-08-03

**Authors:** M. Ángeles Toledano Medina, Jesús Pérez-Aparicio, Alicia Moreno-Ortega, Rafael Moreno-Rojas

**Affiliations:** 1 Instituto de Investigación y Formación Agraria y Pesquera, Avda. Félix Rodríguez de la Fuente sn, Palma del Río, Córdoba, 14700 Andalusia, Spain; 2 Department of Food Science and Technology. Campus Rabanales, Darwin Building, University of Córdoba, 14014 Andalusia, Spain

**Keywords:** black garlic, variety garlic, storage, acidity, reducing sugars, °Brix, polyphenol content, antioxidant capacity

## Abstract

Black garlic is made from the fresh kind, submitting it to a controlled temperature (~65 °C) and humidity (>85 °C) for a prolonged period of time. The aim of this study was to assess the differences in the process and in the final product as a result of employing three garlic varieties (*Spanish Roja, Chinese Spring* and *California White*), and to check the influence of the storage time on fresh garlic in the quality of the final product by using garlic obtained in two different agricultural seasons, that of the current year (2014) and of the previous one (2013). The results revealed some differences in the parameters analysed during the manufacturing of the black garlic from the three varieties used, and even according to the harvest in question. However, when comparing initial and final values of the samples, a very similar evolution in their acidity, reducing sugars, °Brix, pH, polyphenol content, and antioxidant capacity was noted.

## 1. Introduction

The genus Allium belongs to the Alliacae family. Garlic (*Allium sativum*), onion (*Allium cepa*), leek (*Allium porrum*), and chives (*Allium schoenoprasum*) were some more famous herbs from this genus [1].

Black garlic is obtained by a multi-step heating process at a controlled temperature and humidity during a variable period of time from raw garlic [2,3,4,5,6,7]. During the production process, a series of physico-chemical changes of the fresh garlic are produced. The garlic bulbs turn into a darker product, its acidity values increase, it loses its characteristic pungency, and it develops an intense sweet taste. In addition, the total polyphenol content and antioxidant capacity increase during the black garlic process [8]. The final product is characterized by its black colour, sweet-sour taste with a balsamic touch that reminds us of raisins, and for not having the typical garlicky taste that is rejected by many consumers.

This product derived from garlic has been aim for many studies. Some recent publications have described the beneficial effects of black garlic in the prevention or improvement of cardiovascular diseases, diabetes, obesity, or cancerigenous processes, among others [2,3,4]. However, there are few publications providing results on the manufacturing process of this product and especially on the influence of factors like the garlic variety used or the time elapsing after its harvest on the quality of the final product obtained. In keeping with recent studies [9], 70 °C is the temperature of choice for black garlic production process with high relative humidity conditions (80%–90% RH).

The loss of characteristic smell and flavor of fresh garlic is one of the most important quality aspects in black garlic. This loss of fresh garlic flavor largely conditions the duration of the black garlic process, however, if the duration of the process is longer, the acidity values and production costs are higher [8]. The characteristic flavor of fresh garlic is produced when garlic cloves are handled, as sulfur compounds are produced by enzyme alliinase action on S-alk(en)yl substituted cysteine sulfoxides, with S-allylcysteinesulfoxide (alliin) being the most abundant sulfur compound in fresh garlic [10].

Previous studies demonstrated than S-alk(en)yllcysteine sulfoxides content depends mainly on genetic factors and post-harvest storage conditions of garlic bulbs, while the edaphoclimatic conditions during the plant growth have a lower influence [11]. Hornickova et al. (2010) [11] showed a significant increase of S-alk(en)ylcysteine sulfoxides content after a prolonged storage of fresh garlic. However, these results were obtained after eight weeks of storage at 5 °C. Some companies that market fresh garlic carry out storage at −2 °C, in order to keep the garlic in dormant state (dormancy) and to avoid germination of the garlic clove (sprouting). 

In this direction, the aim proposed in this study was to evaluate the employment of three garlic varieties (*Spanish Roja.*, *Chinese Spring* and *California White*) harvested at the same time in two seasons, that of the current year and that of the previous one, and stored at −2 °C after harvest, in the process and in the quality of obtaining black garlic.

## 2. Materials and Methods

To manufacture the black garlic, three fresh garlic varieties were used (*Spanish Roja*, *California White* and *Chinese Spring*) from two different harvests (fresh garlic from the current year and that coming from the previous one). The garlic from the previous year’s harvest was kept under refrigeration at −2 °C up to its processing.

During the black garlic manufacturing process, all the samples were submitted to the same temperature (72 ± 2 °C) and relative humidity (close to 90%) conditions.

Samples of the three varieties were analysed at the beginning and during the manufacturing of the black garlic (at 14, 21, and 34 days of manufacture). In each control, three samples were taken. In each sampling, the following physicochemical properties were determined: soluble solids (°Brix), pH, acidity (% citric acid), content in reducing sugars, total polyphenol concentration, and antioxidant capacity.

### 2.1. Analysis of pH, Soluble Solids (°Brix), Acidity, and Reducing Sugars

The pH analysis was made directly on the ground, homogenized sample using a potentiometer Crison Basic 20 model. To determine the content in soluble solids (°Brix), it was necessary to filter approximately 5 g of the ground sample and the liquid from it in an Abbe refractometer.

Acidity was expressed as % of citric acid. Approximately 15 g of the ground sample was weighed, and sufficient distilled water added to facilitate its homogenization, and next it was filtered for its evaluation with NaOH 0.25 N.

The reducing sugars were determined in accordance with the Rebelein method. Then, 2 mL of the sample homogenized in a beaker of precipitate was taken. To prevent interference from other substances with reducing properties, 16 mL of distilled water, 1 mL of 15% trihydrated potassium hexacyanoferrate (II), and 1 mL of 30% zinc sulphate were added to the 2 mL of sample. This was left in repose for 10 min and 5 mL of this solution was taken, to which 10 mL of 30% potassium iodide, 10 mL of 16% sulphuric acid, and 10 mL of starch paste were next added, after which it was evaluated with sodium thiosulphate 0.55 N, turning a creamy-white colour. The volume employed of thiosulphate in a blank measurement was subtracted from the volume of thiosulphate used and was compared with a calibration line made by titrating with thiosulphate different solutions of glucose in distilled water, thus obtaining the content (g/Kg) of reducing sugars in the sample.

### 2.2. Analysis of Polyphenol Content and Antioxidant Capacity

The samples of each control were lyophilized and 0.3 g of lyophilized sample in 10 mL of a 50% *v*/*v* of ethanol and distilled water was used. It was shaken for one hour in a rotating carousel. It was subsequently filtered using a buchner funnel with a whatman filter over a kitasato flask connected to a vacuum pump. The filtered extract was made up to 25 mL with a 50% *v*/*v* hydroalcoholic solution. Two extractions per each sample were made.

The polyphenol concentration was determined with the Folin–Ciocalteu method [12]. To a 25 mL volumetric flask, 0.5 mL of the extract, 10 mL of distilled water, 1 mL of Folin–Ciocalteu reagent, and 3 mL of 20% *p*/*v* sodium carbonate were added and topped up with distilled water. The mixture was heated at 50 °C for 5 min to accelerate the colouring reaction. Next, it was cooled down with water and a reading at 765 nm in a spectrophotometer was taken. The result was compared with a calibrated curve made by taking solutions of gallic acid of 75, 100, 200, 250, and 300 ppm. The results were expressed by considering the dilution of the sample (0.3 g in 25 mL) in grams of gallic acid equivalent per kg of lyophilized sample.

The antioxidant capacity was determined following the ABTS (2,2’-azino-bis(3-ethylbenzothiazoline-6-sulphonic acid)) radical method [13] and was expressed in mmol equivalent to the Trolox. A total of 2.557 mL was prepared of a solution of the reagent 7 mM ABTS in distilled water, to which 0.333 mL of a solution of 2.5 mM of potassium persulphate in distilled water was added. The solution prepared was stored in darkness for 16 h—the time needed for the formation of the radical (ABTS+). Next, 0.15 mL of the ABTS+ solution was diluted in 15 mL of ethanol and its absorbance value (A_0_) adjusted to 734 nm at 7 min. Into a 1 cm light path cuvette, 0.980 mL of the ABTS + solution and 0.02 mL of the sample extract were placed. This was shaken and the absorbance reading taken at 734 nm and at 7 min (A_1_). The inhibition percentage was calculated by the following expression:

% inhibition = (A_0_ − A_1_) * 100/A_0_.

Subsequently, a calibration curve was drawn with different Trolox concentrations and the inhibition percentage was obtained expressed in mmol equivalent to Trolox per kg of lyophilized sample.

### 2.3. Statistical Analysis

The results obtained were analysed by analysis of variance. A different model for each year was used. Two variation factors were established: the garlic variety with three fixed levels (each of the varieties used in the study), and the control time with four fixed levels (the initial one and each of the controls made at 14, 21, and 34 days of the process). The interaction between both variation factors was included in the model. The analysis of variance was made with a *p* < 0.001 probability error. To determine which levels of each factor presented significant differences, the mean values obtained in the different controls were compared in accordance with Tukey’s test.

## 3. Results and Discussion

Table 1 shows the values calculated of the mean and standard of the mean in the reducing sugars, acidity, °Brix, pH, antioxidant capacity, and polyphenol content. The mean values were grouped according to the control during the black garlic manufacturing process (Initial, 14, 21, and 34 days), depending on the garlic variety used (*Spanish Roja*, *Chinese Spring,* and *California White*) for each year in which the garlic employed was harvested (2013 and 2014).

The result of the analysis of variance was significant for both variation factors in each of the analyses made (acidity, pH, Brix, reducing sugars, antioxidant capacity, and polyphenol content) and in the two harvests in the study.

The crossing factor was only significant in the content of polyphenols in garlic of 2014 owing to, in the two last controls, the different garlic varieties not showing any significant differences.

To explain the results obtained with garlic stored during one-year (2013) in comparison with garlic obtained in the year of the study (2014), one should not discount the edaphoclimatic influences; for this reason, statistical comparison between them was avoided. Although, it is possible and achievable produce black garlic with fresh garlic stored for long periods without finding great differences in the physico-chemical characteristic in the final product. However, there were clear differences of reducing sugars content, mainly for the *California White* variety (0.81 g/kg lyophilized sample for 2014 samples and 2.72 g/kg lyophilized sample for 2013 samples), possibly because this variety could dehydrate more during storage. This also explains the acidity values shown for the *California White* variety at day 0 (0.33% citric acid for 2014 samples and 0.52% citric acid for 2013 samples). Finally, the differences between garlic from both harvests were reduced during the black garlic production process.

### 3.1. Acidity

During the manufacturing process, the acidity value was increasing progressively in all the varieties. The increase could be partly related to derived compounds from browning and heat treatment [14]. These results are in line with previous studies [8,14]. Zhang et al., (2016) [14] showed the acidity values during black garlic production process with different temperatures (60 °C, 70 °C, 80 °C, 90 °C). They reported increasing acidity values for the different process temperatures; if the process temperature was higher, the acidity increase was reached faster. The acidity value reported by Zhang et al. (2016) [14] with 70 °C and 33 to 36 days of the black garlic process was about 4%, similar to the value obtained in our study.

In garlic harvested in 2013, the mean acidity values were significantly higher in the initial control in the var. *Chinese Spring*. The acidity value was also significantly higher in the variety *Chinese Spring* in the controls made at 21 and 34 days. The var. *California White*, in the final control, had a significantly lower acidity than the rest of the varieties, although, in the remaining controls, the differences were not significant compared with those of the var. *Spanish Roja*. In garlic harvested and analysed in 2014, the results were similar, with the var. *Chinese Spring* being the one displaying significantly higher values in the initial and final controls. The var. *California White* gave a significantly lower value in the initial control and at 14 and 34 days (2.79).

The results confirm that, at the temperature established (72 °C), the ideal duration of the process should be under 34 days, as excessive acidity values are reached in all the varieties studied, or that acidity generated in the product should be neutralised. Zhang et al. (2016) [14] reported that an acidity higher than 4% produced an unpleasant acid taste in black garlic. According to our experience, the acidity limit should be around the values of 2% and 2.5%.

**Figure 1:**
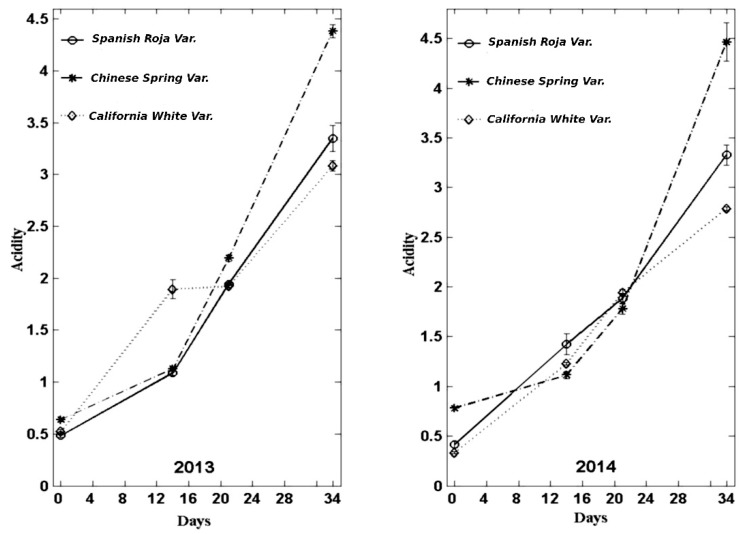
Evolution of acidity (% citric acid) during the manufacture of black garlic with three varieties and two types of garlic: one-year old garlic (2013) and garlic recently harvested (right).

In the Figure 1, the results of the acidity are shown in a graph:

### 3.2. Reducing Sugars

The evolution, the same as in the acidity results, showed an increasing trend from values close to 1 g/Kg up to values close to 30 g/Kg at 34 days. Similar results were reported by other studies with a substantial increase of reducing sugars during the production of black garlic. Choi et al. (2014) [15] showed a 10-fold increase in reducing sugar content during 35 days of maturity stage. Yuan et al. (2016) [16] observed an increase from 187.79% to 790.96%, explained mainly by changes in fructose and glucose content during the process, as black garlic showed similar sucrose concentration to fresh garlic. These considerable increases of fructose and glucose contents during black garlic production could be the result of the hydrolysis of fructans during the process, Yuan et al. (2016) [16] observed a decrease of 84.6% of total fructans content in black garlic in relation to fresh garlic. The increase of reducing sugar content explains the characteristic sweet taste of black garlic.

The reducing sugar content was significantly higher in the var. *California White* with respect to the other varieties in the initial control and at 14 days of the process in garlic of the harvest of 2013. However, in the last control, the *Spanish Roja* variety had a significantly somewhat higher value (31.96 g/Kg). In garlic of the 2014 harvest, the results were lower especially in the final control at 34 days, with the var. *California White* being the one showing a higher value compared with the other varieties. The var. *Chinese Spring* was the one showing the lowest content of reducing sugars out of the three varieties studied in garlic kept from the harvest of 2013 and in garlic of 2014.

**Figure 2:**
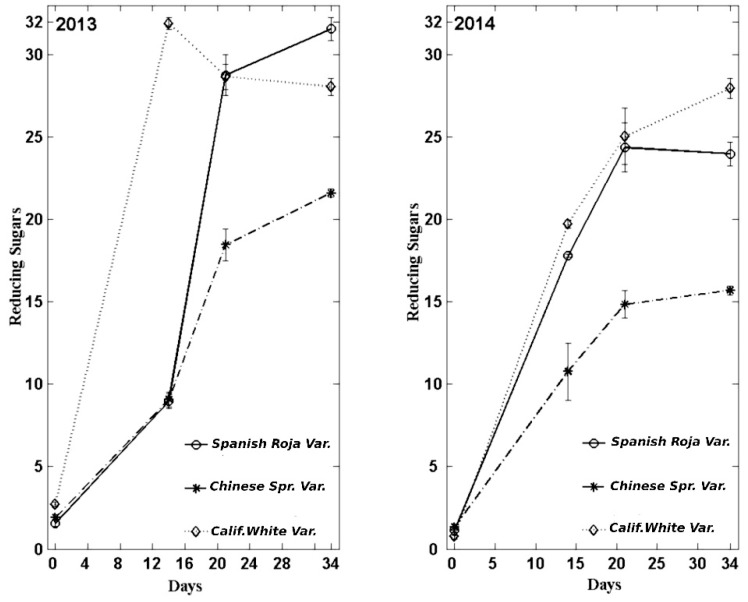
Evolution of the reducing sugar content (g/kg lyophilized sample) during the manufacture of black garlic with three varieties and two types of garlic: garlic aged one year and garlic recently harvested.

In the Figure 2, these results are depicted:

### 3.3. °Brix and pH

The results of °Brix were coherent with the results obtained in reducing sugars, both in garlic kept from the 2013 harvest and analysed in 2014 and in garlic harvested in 2014. Thus, the garlic variety with the lowest value of °Brix was the variety *Chinese Spring* in the initial control and during the whole manufacture process. The results showed higher values in samples from the 2013 harvest in practically all the controls made. Regarding their evolution, they tended to increase during the process.

**Figure 3:**
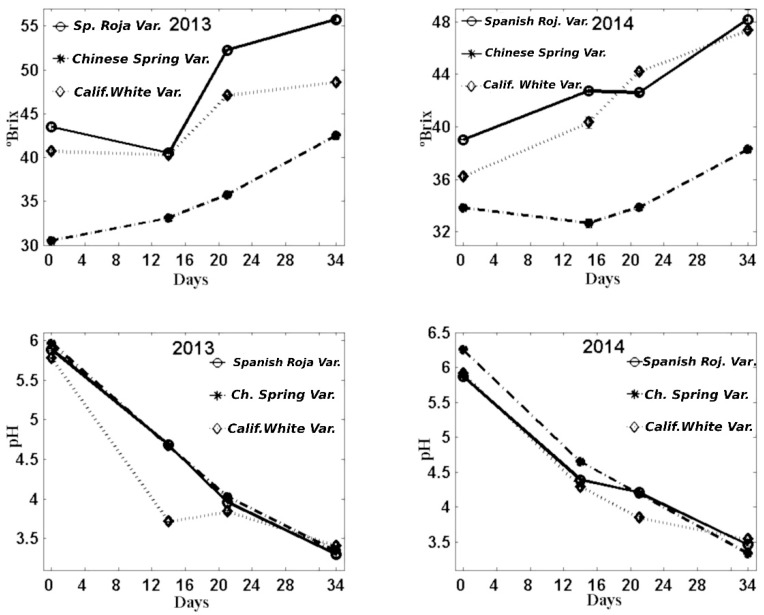
Evolution of °Brix (**top**) and pH (**bottom**) during black garlic manufacture with three varieties and two garlic types: one-year-old garlic (2013) and recently harvested garlic (2014).

The pH results, although they exhibited significant differences between varieties, had a greater similarity between varieties and between both harvests, 2013 and 2014. The Figure 3 shows the results obtained of the °Brix and pH.

Our results about the evolution of °Brix and pH during garlic storage are in line with those reported by Toledano-Medina et al., (2016) [8], Choi et al., (2014) [15], and Bae et al., (2014) [17].

### 3.4. Antioxidant Capacity

Antioxidant capacity evolution showed strong growth up until the maximum reached at 21 days. These values are in line with the results provided by different research works that described a 4.5-fold increase in the initial values [2,8,15]. Choi et al., (2014) [15] also reported lower antioxidant activity during black garlic production, mainly at 21 days of the process. The increase of antioxidant capacity could be related to the increase of total polyphenols, flavonoids, and intermediate compounds of Maillard reaction produced during heat treatment [15,18,19].

The antioxidant capacity results were significantly higher in the var. *Chinese Spring* in all the controls made with garlic from the harvest of 2013 and in the final control of those from the 2014 one. There were no notable differences between either type of garlic (2013 and 2014), and in both cases, a correction in the value obtained in the last control (34 days) with respect to the one made at 21 days was observed.

**Figure 4:**
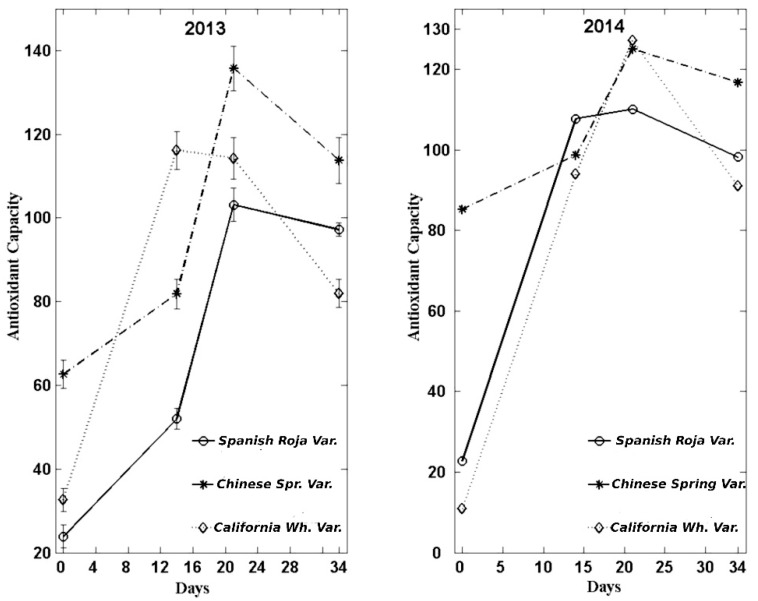
Evolution of antioxidant capacity (TROLOX mmol/kg lyophilized sample) during the manufacture of black garlic with three varieties and two types of garlic: one-year-old and recently harvested.

In the Figure 4, the results obtained are depicted.

### 3.5. Polyphenol Content

In neither of the garlic types (2013 and 2014) were any notable differences seen, and, in this case, no correction in the value obtained in the last control (34 days) was observed. These results are in line with those obtained in previous studies [8,14,15], which showed increased levels of polyphenol content during the black garlic production. In addition, the important increase of the polyphenol content could be related to compounds derived from Maillard reaction, such as 5-hydroxymethylfurfural (5-HMF). Zhang et al. (2016) [14] evaluated the effect of different temperatures during black garlic production on 5-HMF content; they observed a high increase of this compound during the different heat treatments, especially when the temperature was up to 70 °C. Furthermore, Choi et al. (2014) [15] showed an increase of total content of flavonoids during black garlic production, which could explain the increase of total polyphenol content during the process. Lu et al. (2017) [19] reported uridine, adenosine, carbonile alcaloids, and 5-hydroxymethylfurfural as the compounds with higher polyphenolic content and antioxidant capacity in different ethyl acetate extracts of black garlic.

The results were significantly higher in the variety *Chinese Spring* in all the controls made with garlic from the 2013 harvest, although, in the last two controls of the 2014 harvest, the results did not give any significant differences between varieties.

**Figure 5:**
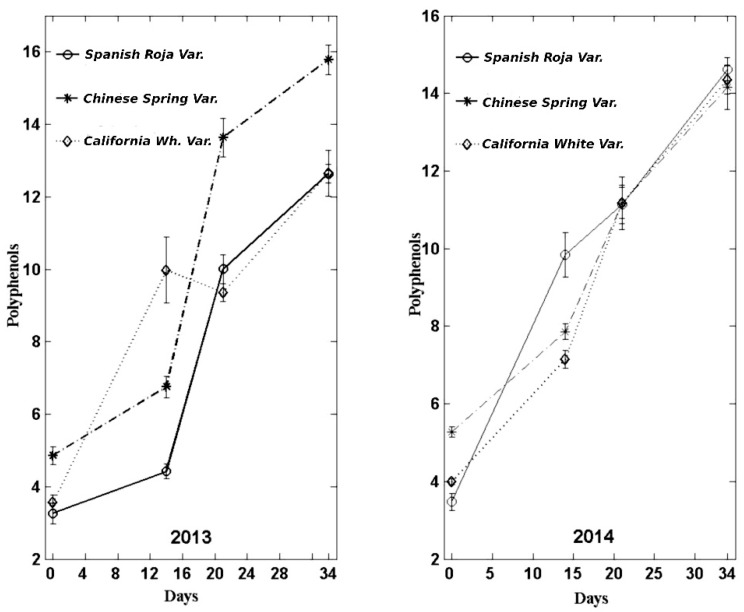
Evolution of the polyphenol content (g Gallic/Kg lyophilized sample) during the manufacture of black garlic with three varieties and two types of garlic: one-year-old and recently harvested.

The Figure 5 shows the results obtained.

## 4. Conclusions

With regard to the manufacturing process, regardless of the variety used and only taking into account the physicochemical parameter evolution, the values obtained were similar to those published by different authors [2,3,4,5,6,8].

In this work, it was demonstrated that black garlic can be made with garlic proceeding from a previous harvest that is kept dormant under refrigeration for one year. This possibility should additionally be reinforced with some procedure in order to differentiate black garlic made with fresh garlic from a recent season from that made with garlic kept from the previous one.

The analyses conducted show some differences between the previous year’s garlic (2013) and that of the study (2014); for example, the 2013 garlic contained a higher content in reducing sugars, °Brix, and acidity, especially for the *California White* variety. The most probable reason for those differences could be a loss of humidity during storage and a resulting concentration.

With regard to the varieties employed, the garlic of the variety *Chinese Spring* exhibited a smaller amount of reducing sugars and °Brix, and a greater acidity than the rest of the varieties analysed in garlic of 2013 and 2014. Its antioxidant capacity and polyphenol content were higher than those of the rest of the varieties, especially in garlic of 2013.

As for the process, it is known that the temperature determines its duration. It should be pointed out that an excessive duration in the process is detrimental to the final product. First, its acidity becomes excessive as the process becomes protracted. Second, the product’s antioxidant capacity diminishes after reaching a prior maximum value when the process is extended, although the polyphenol content goes on increasing.

## Figures and Tables

**Table 1:** Acidity, pH, soluble solids (°Brix), reducing sugars, antioxidant capacity, and polyphenols (grams of gallic acid equivalent per kg of lyophilized sample) of the garlic varieties *Spanish Roja*, *Chinese Spring,* and *California White* in two consecutive harvests.

**Day**	***Acidity***			***pH***			***Brix***		
	***Var. Spanish Roja***	***Var. Chinese Spring***	***Var. California White***	***Var. Spanish Roja***	***Var. Chinese Spring***	***Var California White***	***Var. Spanish Roja***	***Var. Chinese Spring***	***Var. California White***
**2013**									
0	0.48 ± 0.014 a	0.64 ± 0.02 b	0.52 ± 0.02 a	5.88 ± 0.01 a	5.96 ± 0.02 b	5.78 ± 0.01 c	43.5 ± 0.55 a	30.5 ± 0.15 b	40.67 ± 0.18 c
14	1.09 ± 0.02 c	1.13 ± 0.01 c	1.89 ± 0.1 d	4.68 ± 0.01 d	4.66 ± 0 e	3.71 ± 0.01 f	40.5 ± 0.56 c	33.1 ± 0.47 d	40.3 ± 0.1 c
21	1.94 ± 0.01 d	2.19 ± 0.03 e	1.92 ± 0.01 d	3.96 ± 0.05 g	4.03 ± 0.02 g	3.84 ± 0.01 h	52.27 ± 0.07 e	35.77 ± 0.09 f	47.1 ± 0.12 g
34	3.35 ± 0.13 f	4.38 ± 0.06 g	3.08 ± 0.05 h	3.3 ± 0.35 i	3.34 ± 0.2 i	3.4 ± 0.01 j	55.75 ± 0.25 h	42.5 ± 0.5 i	48.6 ± 0.36 j
**2014**									
0	0.42 ± 0.03 a	0.79 ± 0.02 b	0.33 ± 0.01 c	5.87 ± 0.05 a	6.25 ± 0.03 b	5.91 ± 0.02 a	39.03 ± 0.03 a	33.83 ± 0.2 b	36.23 ± 0.19 c
14	1.42 ± 0.11 d	1.11 ± 0.04 e	1.23 ± 0.01 f	4.39 ± 0.05 c	4.65 ± 0 d	4.29 ± 0 e	42.73 ± 0.3 d	32.67 ± 0.35 e	40.33 ± 0.43 f
21	1.88 ± 0.05 gh	1.78 ± 0.06 g	1.94 ± 0.03 h	4.21 ± 0.05 f	4.19 ± 0.01 f	3.85 ± 0.02 g	42.6 ± 0.15 g	33.87 ± 0.3 h	44.17 ± 0.09 i
34	3.33 ± 0.1 i	4.46 ± 0.19 j	2.79 ± 0.02 k	3.47 ± 0.02 h	3.34 ± 0.02 i	3.54 ± 0.01 j	48.17 ± 0.73 j	38.28 ± 0.17 k	47.33 ± 0.08 j
**Day**	***Sugars***			***Antioxidant capacity***			***Polyphenols***		
**2013**									
0	1.57 ± 0.18 a	1.91 ± 0.2 a	2.72 ± 0.14 b	23.9 ± 2.79 a	62.61 ± 3.37 b	32.62 ± 2.76 c	3.26 ± 0.29 a	4.86 ± 0.24 b	3.56 ± 0.2 a
14	8.97 ± 0.45 c	9.07 ± 0.43 c	31.9 ± 0.37*d	52 ± 2.5 d	81.85 ± 3.52 e	116.14 ± 4.49 f	4.42 ± 0.2 c	6.75 ± 0.3 d	9.97 ± 0.91 e
21	28.77 ± 1.23 e	18.46 ± 0.99 f	28.66 ± 0.76 e	103.11 ± 4.0 g	135.74 ± 5.37 h	114.31 ± 4.98 f	10 ± 0.4 e	13.64 ± 0.52 f	9.36 ± 0.25 e
34	31.56 ± 0.69 d	21.61 ± 0.25 g	28.01 ± 0.51 e	97.21 ± 1.6 i	113.77 ± 5.49 f	81.99 ± 3.32 e	12.63 ± 0.26 g	15.79 ± 0.41 h	12.65 ± 0.64 g
**2014**									
0	1.17 ± 0.33 a	1.34 ± 0.17 a	0.81 ± 0.20 b	22.75 ± 2.25 a	85.22 ± 5.6 b	10.97 ± 1.42 c	3.48 ± 0.22 a	5.28 ± 0.13 b	4 ± 0.08 c
14	17.8 ± 0.1 c	10.77 ± 1.73 d	19.72 ± 0.26 e	107.68 ± 3.39 d	98.71 ± 4.2 b	94.07 ± 5.17 b	9.84 ± 0.57 d	7.86 ± 0.2 e	7.15 ± 0.23 f
21	24.37 ± 1.48 f	14.85 ± 0.83 g	25.05 ± 0.71 f	110.01 ± 3.68 d	125 ± 5.7 e	127.17 ± 2.89 e	11.14 ± 0.5 g	11.17 ± 0.4 g	11.17 ± 0.68 g
34	23.98 ± 0.71 f	15.7 ± 0.28 g	27.96 ± 0.62 h	98.21 ± 7.82 d	116.7 ± 4.4 e	90.99 ± 6.85 d	14.62 ± 0.29 h	14.16 ± 0.58 h	14.34 ± 0.36 h

0: initial control; 14: control at 14 days; 21: control at 21 days; 34: control at 34 days. Different letters (two-ways ANOVA) denote significant differences among the garlic variety with three fixed levels and the control time with four fixed levels for the same parameter.
